# Blue light regulates conidia production in *Shiraia bambusicola* via calcium/calmodulin signaling

**DOI:** 10.1186/s13568-026-02036-2

**Published:** 2026-03-07

**Authors:** Wen Du, Chunlong Sun, Shuai Shang, Wang Li, Wenwen Huang, Ran Wang, Jian Li, Guolan Liu, Hongguo Wang, Chenzhao Wang, Zhiwei Su, Qian Yang

**Affiliations:** 1 College of Biological and Pharmaceutical Engineering, Shandong University of Aeronautics, Binzhou, 256600 China; 2 Binzhou key laboratory of Chemical Drug R&D and Quality Control, Binzhou, 256600 China

**Keywords:** *Shiraia bambusicola*, Conidial production, Blue light, Ca^2+^ signalling

## Abstract

*Shiraia bambusicola* is a valuable medicinal fungus parasitic on bamboo, and extracting its active substances represents a sustainable resource utilization strategy. Conidial production is crucial for in the industrial utilization and growth of *S. bambusicola*. Since light significantly affects its development, we investigated how different light conditions influence conidial production. Blue light induced conidia production 6 days earlier than darkness and increased yield by 7.04-fold, accompanied by thickened mycelia with little change in conidial morphology. Proteomic analysis of blue light-induced conidiation identified 455 differentially expressed proteins (DEPs), and most of them were down-regulated. Gene Ontology (GO) annotation results of DEPs showed that the biological process of *S. bambusicola* conidiation is complex, and the carbohydrate metabolic process is the most significant one. Kyoto Encyclopedia of Genes and Genomes (KEGG) metabolic pathway and Clusters of Orthologous Groups (COG) database analysis showed that carbohydrate transport and metabolism, and phagosome pathway may be mainly involved. Blue light treatment significantly changed the abundance of calcium ion binding (CIB), phospholipase (PL), calreticulin (CRT), calnexin (CANX). The addition of calcium ions (Ca^2+^) inhibitors BAPTA-AM significantly affected conidial production, as well as the relative contents of calcium/calmodulin-dependent protein kinase (*CaMK*), *CANX*, and glycoside hydrolase (*GH*). In contrast, supplementation with Ca^2+^ and LaCl_3_ had no significant effect on the conidial production. The results showed that Ca^2+^ was involved in the conidiation, primarily originating from intracellular calcium pools. This study supports the use of blue light in industrial conidial production and provides proteomic insights into the molecular mechanisms of conidia formation, highlighting the role of Ca²⁺ in fungal development.

**Supplementary Information:**

The online version contains supplementary material available at 10.1186/s13568-026-02036-2.

## Introduction

*Shiraia bambusicola* Henn., an obligate parasitic fungus on bamboo plants, is primarily distributed south of the Yangtze River in China and in Japan. Its host range encompasses bambusoideous plants such as *Phyllostachys*, *Pleioblastus*, and *Brachystachyum* (Ren et al. 2022). The fungal fruiting body “Zhuhuang” formed by this fungus is included as a traditional Chinese medicinal material in the *Chinese Pharmacopoeia* (Dong et al. 2021; Ren et al. 2022). It exhibits composite pharmacological activities including cough suppression, pain relief, muscle relaxation, and collateral activation, holding significant application value in the clinical treatment of rheumatoid arthritis, chronic bronchitis, and neurological disorders (Yu and Fischer 2019). Modern pharmacological studies indicate that the medicinal value of *S. bambusicola* stems from its unique secondary metabolite profile, primarily comprising hypocrellin-based photosensitizers, immunomodulatory polysaccharides, mannitol, and organic acids. Among these, hypocrellins achieve a dual effect in photodynamic therapy: selective tumor cell killing combined with drug-resistant bacterial inhibition (Dong et al. 2021; Ren et al. 2022; Bao et al. 2023). This characteristic sustains international interest in its potential for novel anticancer and antibacterial drug development. Its mechanisms of action and clinical applications represent a frontier in natural medicinal chemistry research (Dong et al. 2021; Bao et al. 2023). In recent years, increasing market demand and excessive harvesting have led to a progressive decline in wild *S. bambusicola* resources. Coupled with the slow growth of natural fruiting bodies and persistently high market prices, artificial cultivation techniques have become a key research focus. Advances continue to emerge as research into *S. bambusicola* and its active components deepens. Current research focuses on active substances produced by *S. bambusicola*, such as hypocrellins, anthraquinones, laccases, and polysaccharides. The primary aims include their isolation, purification, and optimization of culture medium composition and cultivation conditions (Shen et al. 2023).

Among numerous environmental factors, light is crucial for the growth and development of organisms (Yu and Fischer 2019). In their natural habitats, the fruiting bodies of *S. bambusicola* are perpetually exposed to seasonal temperature and ultraviolet radiation stress. To adapt to this environment, fungi have evolved corresponding survival strategies, including reducing lipid and DNA oxidative damage and producing stress-resistant spores (Dijksterhuis 2019). As a daily environmental factor, light cannot be ignored in the growth and development of macrofungi. Different species of macrofungi respond variably to visible light wavelength, intensity, and quality, and their requirements change with developmental stages. Light can regulate various life activities in fungi (including ascomycetes and basidiomycetes), such as growth, phototropism, and circadian rhythms (Luo et al. 2020). For example, in the ascomycete *Monascus purpureus*, darkness promotes red pigment synthesis, while direct sunlight completely inhibits its production (Babitha et al. 2008). Alternating light and dark treatments can induce conidiophore formation and increase conidial production in *Alternaria solani* (Zhao et al. 2021). Light not only regulates fungal asexual growth and metabolite synthesis but also affects their sexual development (Guo et al. 2015). Light signal perception is widespread in animals, plants, and fungi, involving various photoreceptor proteins (Babitha et al. 2008; Guo et al. 2015; Zhao et al. 2021). Research on fungal photoreceptors has primarily focused on *Neurospora crassa*, with explorations also in *Mucor*, *Trichoderma*, *Phycomyces*, and *Aspergillus*. Three classes of fungal photoreceptor proteins have currently been identified: blue-light receptors containing light-oxygen-voltage **(**LOV) domains; red-light receptors represented by phytochromes; and rhodopsins (Waschuk et al. 2005). The photoreceptors more commonly reported in macrofungi are blue-light receptor proteins, such as white collar-1 (WC-1) and white collar-2 (WC-2), and their homologs. With the continuous enrichment of macrofungal genomic data, the prospects for discovering new photoreceptor encoding genes are broad (Guo et al. 2015; Galindo et al. 2022).

Regarding *S. bambusicola* specifically, environmental factors hold significant ecological importance. Research indicates that culturing under light/dark cycles combined with 32 °C can significantly increase hypocrellin A (HA) production by 73–200% (Wen et al. 2022). Field observations also confirm that the occurrence of *S. bambusicola* fruiting bodies on host bamboos is closely related to light intensity and temperature, with light being a key factor in determining the timing and yield of fruiting body formation (Dong et al. 2021; Ren et al. 2022; Wen et al. 2022). Although significant progress has been made in the cultivation research of *S. bambusicola*, a gap remains before large-scale industrial production can be achieved. It is noteworthy that the optimal habitat for fungi to accumulate secondary metabolites often differs from, or may even be opposite to, the conditions required for their growth and development. For instance, the white-rot fungus *Phanerochaete chrysosporium* produces higher yields of lignin peroxidase under green light, while its sporulation is often associated with stress conditions (Zhang et al. 2016). Research on related mechanisms also supports this view: Although the monocarboxylate transporter gene *MpMch2* positively regulates conidial and ascospore development in *M. purpureus*, it has little effect on the production of monascus pigments or γ-aminobutyric acid (Pan et al. 2024). The mitogen-activated protein kinase (MAPK) signaling pathway enables *A. alternata* to recognize and respond to physicochemical signals on pear fruit skin by regulating spore germination and appressorium formation (Liu et al. 2024). In bioproduction industries, conidia serve as the starting material for seed culture and are a crucial link in achieving large-scale fungal production. However, research on *S. bambusicola* conidia remains relatively scarce. Enhancing the conidiation capacity of *S. bambusicola* is of significant importance for reducing production costs, investigating its relationship with active compound production, controlling contamination, and strain preservation (Guo et al. 2015). Therefore, in-depth exploration of methods to increase conidial yield and their regulatory mechanisms represents an effective approach to overcome bottlenecks in the industrial application of *S. bambusicola*.

Research on the conidiation mechanism of *S. bambusicola* holds considerable value. Our research group has previously conducted preliminary investigations in this area (Du et al. 2024). Nevertheless, the effects of different light spectra on *S. bambusicola* conidiation and the underlying signaling pathways remain unreported. To deeply analyze the environmental factors, particularly light spectra, required for conidiation, this study systematically investigated changes in conidial yield and mycelial morphological responses of *S. bambusicola* under different light conditions. Furthermore, enzymatic digestion coupled with mass spectrometry was employed for differential proteomic analysis of the conidiation process under the influence of light spectra, aiming to elucidate the molecular mechanisms underlying enhanced conidial production. The significance of this study lies in two aspects: On one hand, it can provide a theoretical basis for introducing scientific light-regulation strategies into the artificial cultivation and industrial production of *S. bambusicola*. On the other hand, it will help establish optimized processes for increasing conidial yield and provide novel insights into the key metabolic pathways and regulatory molecules involved in the conidiation process.

## Materials and methods

### Strains, culture media, and chemicals

*Shiraia bambusicola* BZ16 was isolated and identified by the Biopharmaceutical Teaching and Research Section of Shandong University of Aeronautics. It is preserved in the China Center for Type Culture Collection under preservation number CCTCC NO: M209141. The modified potato dextrose agar (PDA) media comprised 50 g of tender stems and leaves from *Pleioblastus amarus*, 50 g of potato, 10 g of glucose, 18 g of agar, and 1,000 mL of distilled water. The experiment employed 9 cm diameter Petri dishes for culturing at 25 °C. Protein and DNA extraction reagents were purchased from Tiangen Biotech, and all other chemicals and reagents used were of analytical grade.

### Lighting system design

The lighting system employs LuoPu PGC300 units equipped with four light sources: a red light source with a peak wavelength of 650 nm and full width at half maximum (FWHM) of 20 nm; a green light source peaking at 520 nm (FWHM 15 nm); a blue light source at 460 nm peak wavelength (FWHM 20 nm); and a white light source delivering continuous spectrum coverage from 400 to 700 nm. Petri dishes were positioned on a temperature-controlled platform maintained at 25.0 °C. A vertical distance of 30 cm between the light source and samples ensured uniform illumination across the exposure area. Light intensity was fine-tuned to achieve a photon flux density of 50 µmol/m²/s at the sample surface. A dark control group was simultaneously processed under identical conditions without light exposure.

### Conidia harvesting

The BZ16 strain was inoculated on the modified PDA medium and cultured under dark and blue light for 12 days. Mycelia cultured in the dark (SC) and in blue light (SB) were collected as the control group and the experimental group, the proteome was tested again. Conidia were counted according to the method of Zhu et al. (2021). For solid culture, add a 0.02% Tween-80 solution to the test plates and mix thoroughly. Solutions containing spores were obtained and counted with hemocytometer under the light microscope to calculate the average amount of conidiospore contained in each Petri dish. For ease of comparison, the data can be converted to the number of conidia per unit area (conidia/cm²) based on standard laboratory Petri dishes with an inner diameter of 8.5 cm.

Morphological Observation and Measurement: The cultural characteristics of *S. bambusicola* under various conditions were systematically described and recorded. The conidia were stained with lactophenol cotton blue (LPCB) and counted using a hemocytometer under a light microscope. Scanning electron microscopy (SEM) was employed for the observation and imaging of *S. bambusicola* conidia and hyphae.

### Experiments with different culture conditions

Mycelial plugs were transferred to the center of modified PDA plates, cultured in darkness at 25 ℃ for 4 days, and then under darkness, red light, blue light, green light and white light from the fifth day after inoculation. Each culture condition was replicated five times. At 4, 6, 8, 10, and 12 days post-inoculation (i.e., 0, 2, 4, 6, and 8 days after initiating light exposure), colony diameter and conidial count of *S. bambusicola* in each Petri dish were determined.

The subcultured mycelia pieces were inoculated on the modified PDA medium for 4 days. 1.5 mL of 5 µmol/L BAPTA-AM, 20 µmol/L LaCl_3_ and 5 mmol/L CaCl_2_ were uniformly added to the culture dish (Qin et al. 2019; Liu et al. 2023; Locke et al. 2024), and then cultured under blue light for 6 days.

### Protein extraction and preparation

In this study, samples of hyphae undergoing conidiation of *S. bambusicola* cultured under blue light illumination and dark conditions for 10 days were collected. The sampling area was uniformly centered at a point 2.0 cm away from the inoculation site, covering a circular region with a diameter of 1.0 cm where hyphae bearing conidia were present. Equal weights of the hyphae undergoing conidiation from the two aforementioned culture conditions were taken for subsequent experiments.

The mycelial sample was homogenized in a lysis buffer containing 4% SDS and denatured by heating at 95 °C for 5 min, followed by ultrasonication (Branson Sonifier, 10 min, 40% amplitude). After centrifugation (12,000 × g, 15 min, 4 °C), proteins in the supernatant were precipitated using trichloroacetic acid (TCA, final concentration 20%), washed three times with pre-cooled acetone, and air-dried. The protein pellet was dissolved in 8 M urea/100 mM Tris-HCl buffer (pH 8.0). Protein concentration was determined using the BCA method (Pierce™). Prior to digestion, proteins were reduced with 10 mM dithiothreitol (DTT, 37 °C, 1 h) and alkylated with 40 mM iodoacetamide (IAA, room temperature, in the dark, 30 min). Subsequently, the urea concentration was diluted to below 2 M with 100 mM Tris-HCl (pH 8.0). Sequencing-grade modified trypsin was added at an enzyme-to-substrate ratio of 1:50 (w/w), followed by incubation at 37 °C with shaking for 12 h. Digestion was terminated by adding trifluoroacetic acid (TFA) to a final concentration of 0.1% and adjusting the pH to approximately 6.0. The resulting peptide mixture was desalted using a Sep-Pak C18 column (Waters) according to the manufacturer’s instructions, dried in a vacuum concentrator (Eppendorf), and stored at -80 °C until LC-MS/MS analysis.

### MS detection and database search

LC-MS/MS analysis was performed using a TripleTOF 5600 + mass spectrometer (SCIEX) coupled with an Eksigent microLC 415 nano-HPLC system (SCIEX). The desalted peptides were reconstituted in the loading solvent (2% acetonitrile, 0.1% formic acid) and loaded onto a C18 trap column (5 μm, 300 Å, 300 μm × 5 mm). Peptide separation was carried out on a C18 analytical column (3 μm, 120 Å, 300 μm × 150 mm) using a 90-minute linear gradient, with mobile phase B increasing from 5% to 35% (mobile phase A: water containing 0.1% formic acid; mobile phase B: acetonitrile containing 0.1% formic acid), at a constant flow rate of 5 µL/min. Statistical analysis was performed to evaluate the mass deviation of the identified peptides across all samples. The distribution of peptide mass deviations fell within ± 20 ppm, demonstrating excellent MS accuracy (Fig. S1). Most peptides had no missed cleavage sites, indicating comprehensive enzymatic digestion and meeting the required standards for sample preparation (Fig. S2). MS data generated by TripleTOF 5600 + were retrieved through ProteinPilot. The self-built proteome reference database was used for searching. Unused ≥ 1.3 was used as the standard to screen qualified protein information, and the remaining identification information was used for subsequent analysis. The differential proteome (*P* ≤ 0.05 and SC/SM Fold change (FC) ≥ 1.5 or ≤ 0.067) of conidial production under suitable conditions of the BZ16 strain was obtained. The annotation of proteins mainly used the Uniprot database (https://www.uniprot.org/) and the NCBI blast database.

GO enrichment analysis, KEGG pathway (http://www.kegg.jp/) enrichment analysis, and COG database (http://eggnogdb.embl.de/) classification analysis were performed on the screened differentially expressed proteins (Locke et al. 2024). The significance of enrichment was assessed using the hypergeometric test. Specifically, with all identified proteins as the background set, the test evaluated whether the differentially expressed proteins were significantly enriched in specific functional categories or pathways. P values represent the probability of observing the enrichment of differentially expressed proteins in a given category by chance. A significance threshold of *P* < 0.01 was applied, and the most significantly enriched terms were selected for presentation.The mass spectrometry proteomics data have been deposited to the ProteomeXchange Consortium (https://proteomecentral.proteomexchange.org) via the iProX partner repository (Chen et al. 2022) with the dataset identifier PXD070936. Proteomic MS detection and data analysis were all completed in cooperation with Sangon Biotech (China).

### Quantitative PCR (qPCR) analysis

The equal weight experiment was carried out with two morphologies of *S. bambusicola*. The procedure is repeated five times for each sample. Primers of differentially expressed genes were designed using online tools and softwares (Table S1). Total RNA was extracted and reverse-transcribed into cDNA. Specific primers and SYBR Green i were used for qPCR. 18 S rRNA gene was used as internal reference gene. The cycle parameter was set at 95℃ for 3 min, and then it was cycled 40 times, at 95℃ for 30 s, 57℃ for 30 s, and 72℃ for 15 s. The relative gene expression was calculated by 2^−ΔΔCt^. The differential expression of glycoside hydrolase (*GH*), calnexin (*CANX*) and calcium/calmodulin-dependent protein kinase (*CaMK*) genes were selected to analyze the increased production of *S. bambusicola* conidia under light conditions (Shen et al. 2023).

### Data analysis

Statistical analyses in this study were conducted based on the respective data types. All analyses were performed using SPSS 24.0 and Origin 2022 software. For conventional experiments (excluding proteomics data), results are expressed as mean ± standard deviation, with each group consisting of 3–5 independent replicates. Comparisons between two groups were performed using Student’s *t*-test, while comparisons among multiple groups were analyzed by one-way analysis of variance (ANOVA), followed by appropriate post-hoc tests when necessary. In addition, Pearson correlation analysis was applied to part of the data. The statistical significance level was set at *P* < 0.05.

## Results

### Effect of different light conditions on the conidial production

**Fig. 1:**
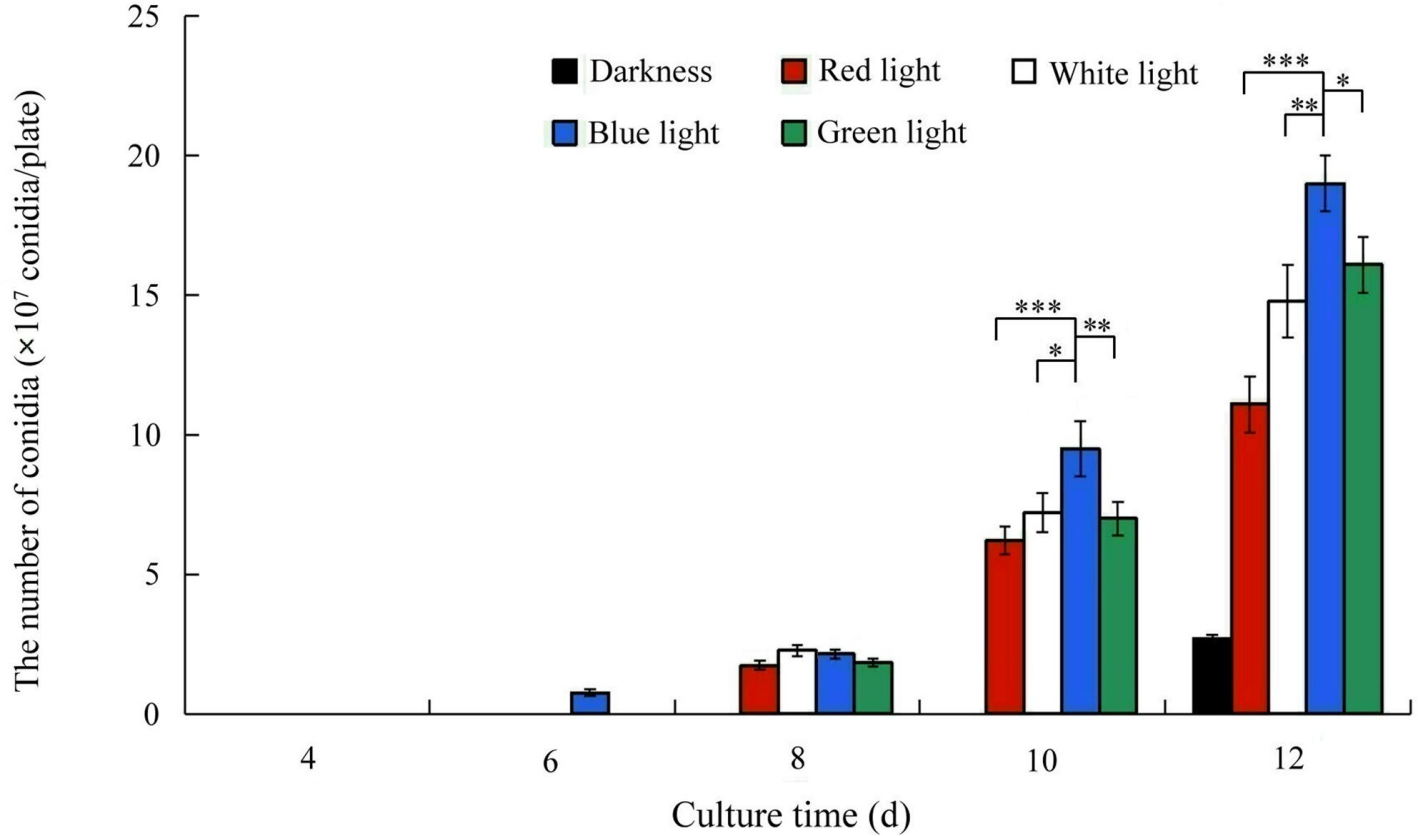
The effects of different light illumination on conidial production.Values were means of five independent experiments. Bars represented standard errors. *P < 0.05;**P < 0.01; ***P < 0.001

**Fig. 2:**
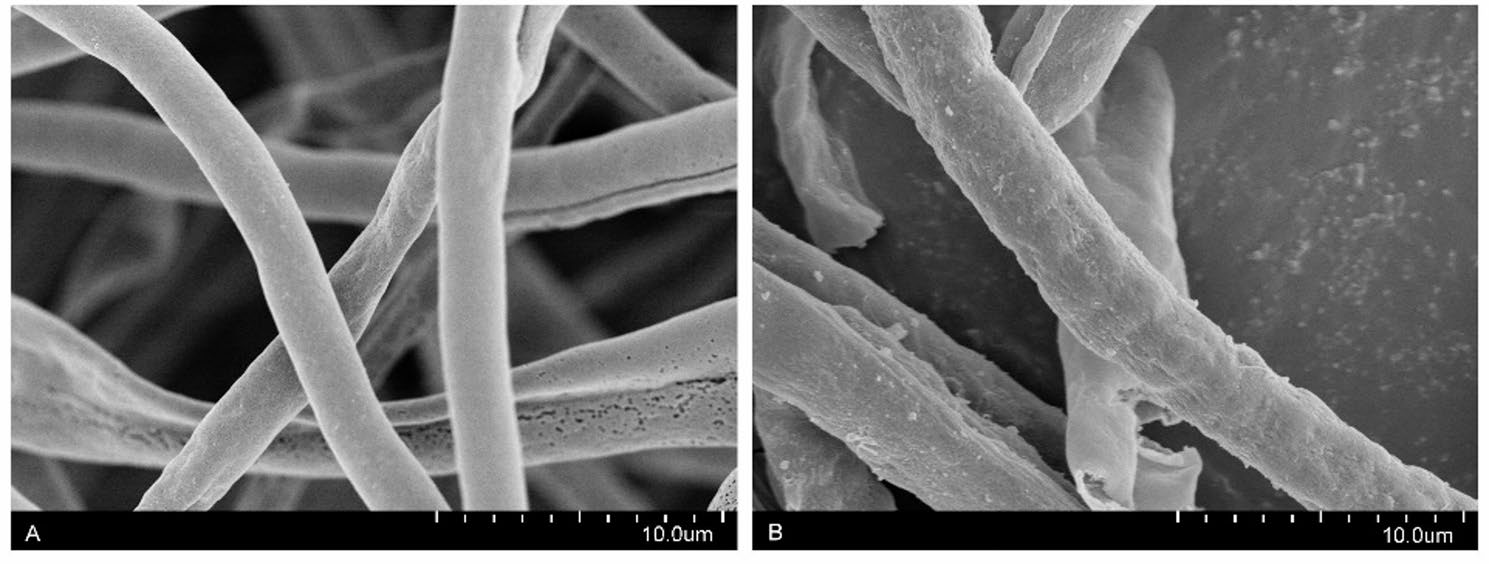
Scanning electron microscopy images of mycelia: **A** Control mycelia **B** Mycelia under blue light. Scale = 10 μm

Using darkness as a control, this study examined the effects of blue, red, and white light on conidial production in *S. bambusicola* (Fig. 1). The results indicated that all three light promoted conidial production throughout the experiment. By day 10, the highest conidial production under blue light treatment reached 1.67 × 10⁶ conidia/cm², followed by green light, white light. The lowest conidial production was observed under red light, with no conidial production in darkness. Significant changes in the conidial production, biomass, and morphology of *S. bambusicola* were observed under blue light. By day 5, hyphae completely colonized the plate. Conidial production subsequently increased to 3.35 × 10⁶ conidia/cm² by day 12 (Fig. 1), showing a significant difference compared to the control group (*p* < 0.01). Under different light conditions, the mycelial morphology of *S. bambusicola* changed, and the timing of producing conidia differed. The time required for conidial production was 6 days under blue light compared with 12 days in darkness (Fig. 1). Conidia size did not significantly change (Fig. S3). The width of mycelia, measured under a microscope, was 4.9–5.8 μm under blue light, whereas it was 3.2–3.5 μm under darkness (Fig. 2). These findings indicate that blue light promotes the mycelial growth and condial formation of *S. bambusicola.*

### Basic proteomic identification information

In this study, the differentially expressed proteins (DEPs) of blue light in conidia formation of *S. bambusicola* were analyzed. We performed protein separation on the samples and identified a substantial number of proteins. The total number of spectra was 86,700, a comparison with a custom protein database identified 1987 proteins. The ratio of the number of spectra between different sample groups and the average number of spectra were calculated. The scatter distribution of proteins was annotated to indicate significant differences. A total of 455 DEPs were identified between the SC and SB groups (*P* < 0.05 and SB/SC fold change (FC) > 2.0), including 169 upregulated and 286 downregulated proteins.

### GO functional enrichment analysis

**Fig. 3:**
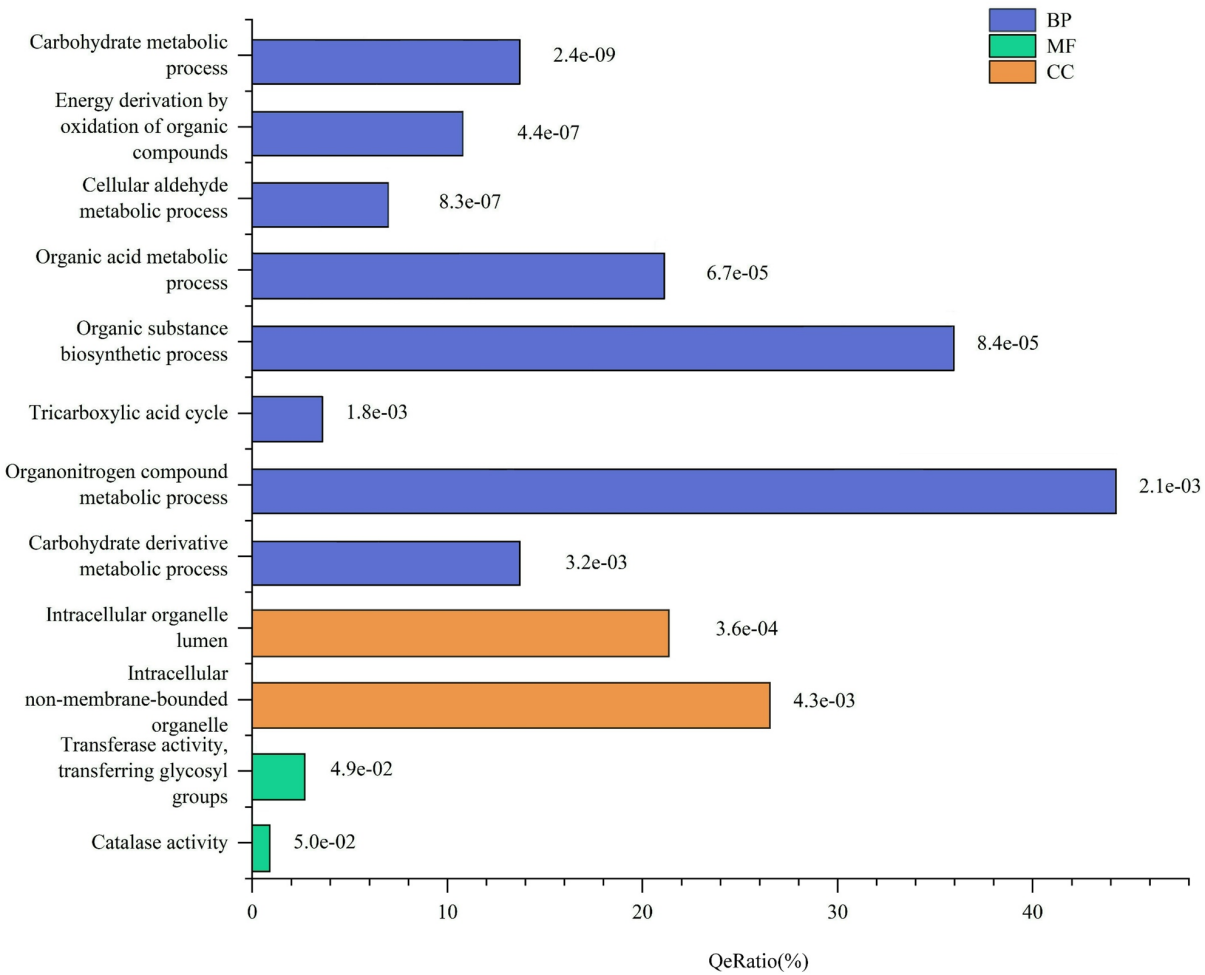
GO analysis and enrichment results of DEPs. The number after Bar represents the P-value of the enrichment analysis. The y-axis represents the top 12 GO terms for MF, BP, and CC. The x-axis indicates QeRation, which is the ratio of the number of differentially expressed transcripts for a GO term to the total number of DEPs

The results of the GO enrichment analysis for DEPs during the process of conidial production under blue light in *S. bambusicola* strain BZ16 are presented in Fig. 3. Comprehensive analysis of proteins within the biological process (BP), molecular function (MF), and cellular component (CC) categories revealed that the BP category had the greatest number and significance of pathways (Fig. 3). Among the DEPs involved in BP under blue light, significant differences were mainly concentrated in carbohydrate metabolic process, energy derivation by oxidation of organic compounds, cellular aldehyde metabolic process, organic acid metabolic process, and organic substance biosynthetic process. Among these, the carbohydrate metabolic process demonstrated the strongest significance in protein enrichment (*P* = 2.4E-09).

Regarding the cellular localization of the DEPs related to conidial formation in *S. bambusicola*, a diverse distribution was observed in CC. The most significant enrichment was found in the intracellular organelle lumen (*P* = 0.000361), which includes various proteasome subunit alpha types involved in the nitrogen compound metabolic process and so on (Fig. 3). GO analysis revealed that these significant proteins are predominantly located in intracellular non-membrane-bounded organelles. The DEPs accounted for 35.98% of the total proteins and were primarily involved in ATP signaling regulation, calmodulin, transmembrane transport, protein localization and folding, signal protein elongation, transport factors, molecular chaperones, and other processes. The results of the MF analysis indicate that the DEPs primarily participate in activities such as transferase activity, transferring glycosyl groups, and catalase activity. Notably, the most significantly enriched term was transferase activity, transferring glycosyl groups (*P* = 0.0488). This suggests that oxidative stress may play a role in the process of the conidiation. Within this pathway, we identified four catalase-peroxidases (katGs): A0A1Y2LX81, A0A178DXJ7, A0A6A5RHX9, and A0A6A6ZDW5. Under blue light, the expression of A0A1Y2LX81 was upregulated in *S. bambusicola* conidia (SB/SC fold change = 3.00), while the other three (A0A178DXJ7, A0A6A5RHX9, and A0A6A6ZDW5) were downregulated, with SB/SC values of 3.46, 3.70, and 1.00, respectively. This indicates that these katGs may belong to two distinct types and potentially serve different oxidative functions.

### KEGG pathway and COG database enrichment analysis

**Fig. 4:**
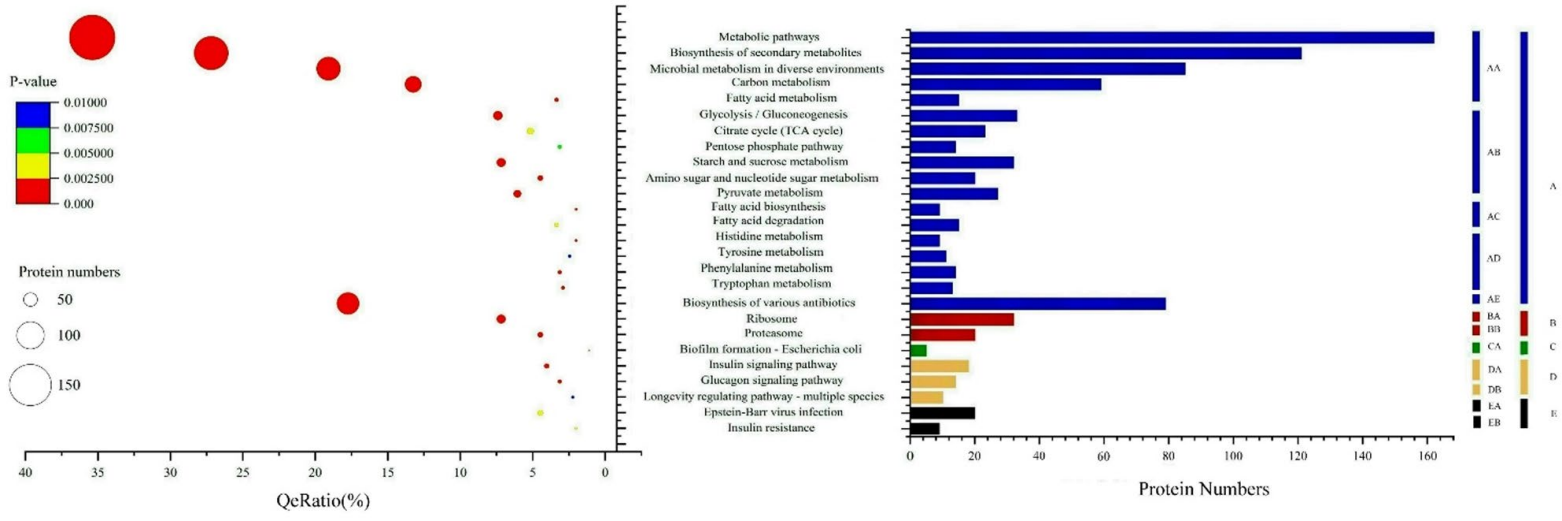
The KEGG pathway analysis and enrichment results of DEPs. **A** Metabolism, *AA* Global and overview maps, *AB* Carbohydrate metabolism, *AC* Lipid metabolism, *AD* Amino acid metabolism, *AE* Biosynthesis of other secondary metabolites, **B** Genetic Information Processing, *BA* Translation, *BB* Folding, sorting and degradation, **C** Cellular Processes, *CA* Cellular community-prokaryotes, **D** Organismal Systems, *DA* Endocrine system, *DB* Aging, **E** Human Diseases, *EA* Epstein-Barr virus infection, *EB* Insulin resistance. The y-axis represents the top 26 KEGG pathways terms. The x-axis indicates the number of DEPs and QeRation. The color key attached to the bubble diagram indicates enrichment significance (P-value). Bubble size represents the number of DEPs in respective pathways

Pathway analysis is a crucial and direct approach for obtaining a comprehensive understanding of the biological processes and characteristics of cells. KEGG pathway annotation and enrichment analysis identified 44 secondary pathways that were significantly enriched (*P* < 0.05). Among these pathways, 25 secondary pathways were selected at a threshold of *P* < 0.01. Figure 4 displays these 25 KEGG secondary pathways, which fall into five major classification levels within the KEGG database: Metabolism, Genetic Information Processing, Cellular Processes, Organismal Systems, and Human Diseases. During the growth process of *S. bambusicola* conidia, a considerable number of proteins associated with signal transduction pathways were expressed. The majority of DEPs were involved in metabolic processes, primarily promoting gene expression centered around carbon metabolism. Changes in growth and development primarily involve various nutrients and hormone signals necessary for conidial formation. The five secondary pathways with the greatest enrichment of DEPs were biosynthesis of secondary metabolites, microbial metabolism in diverse environments, starch and sucrose metabolism, glucagon signaling pathway, and insulin signaling pathway. Metabolic pathways had the greatest number of DEPs, with significant *P* values for biosynthesis of secondary metabolites, indicating a broad metabolic distribution of proteins.

**Fig. 5:**
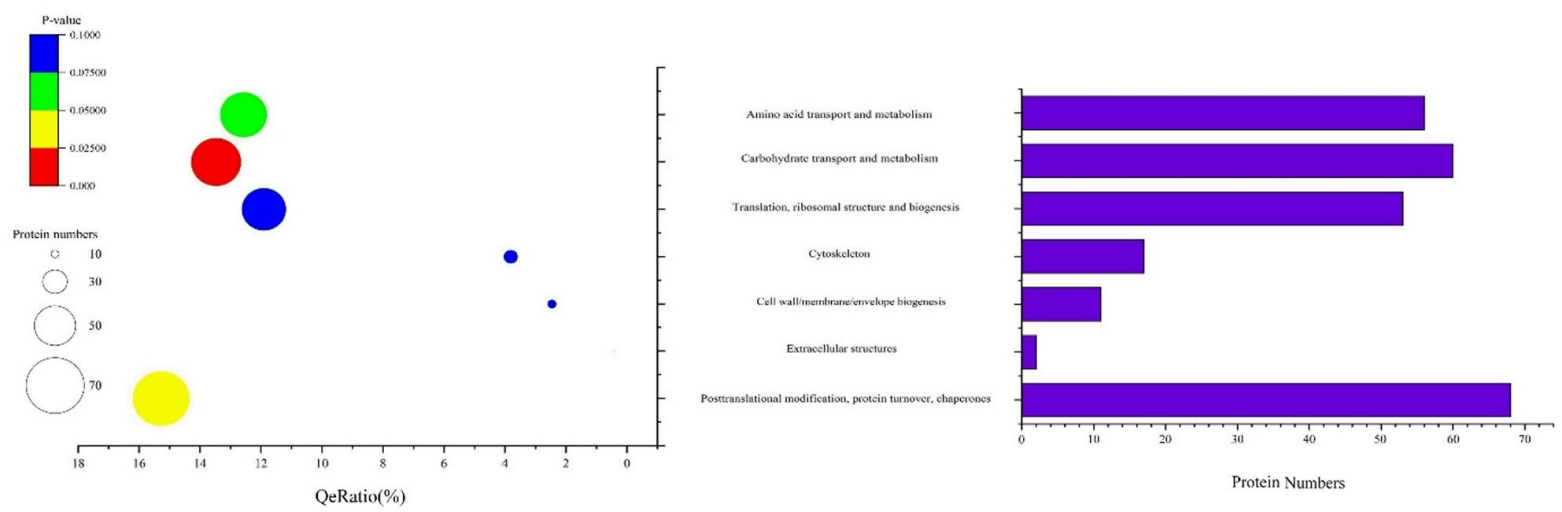
The COG analysis and enrichment results of DEPs. The y-axis represents the top 7 COG pathways terms. The x-axis indicates the number of DEPs and QeRation. The color key attached to the bubble diagram indicates enrichment significance (P-value). Bubble size represents the number of DEPs in respective pathways

In the analysis using the Clusters of Orthologous Groups (COG) database, the DEPs in conidia under blue light were sorted into 23 major functional groups. The categories with the highest representation were posttranslational modification, protein turnover, chaperones, carbohydrate transport and metabolism, amino acid transport, and metabolism closely followed. Notably, proteins involved in carbohydrate transport and metabolism were the most significantly enriched (*P* < 0.001, Fig. 5). In addition, proteins related to post-translational modification, protein turnover, and chaperones also showed significant enrichment (*P* = 0.0283). Comprehensive analysis of the data revealed a prominent concentration of DEPs involved in carbohydrate transport and metabolism. Within this category, significant numbers of proteins were found to be involved in starch and sucrose metabolism, galactose metabolism, fructose and mannose metabolism, and phenylpropanoid biosynthesis. Among the identified proteins, 19 had unknown functions, as did some proteins with known functions for which their specific metabolic localization has yet to be determined. As bioinformatics continues to advance, an increasing array of computational methods and technologies will be applied to further investigate these predicted proteins, allowing for enhanced accuracy in determining the metabolic pathways in which the DEPs are located.

### Proteins related to Ca^2+^ signaling

**Fig. 6:**
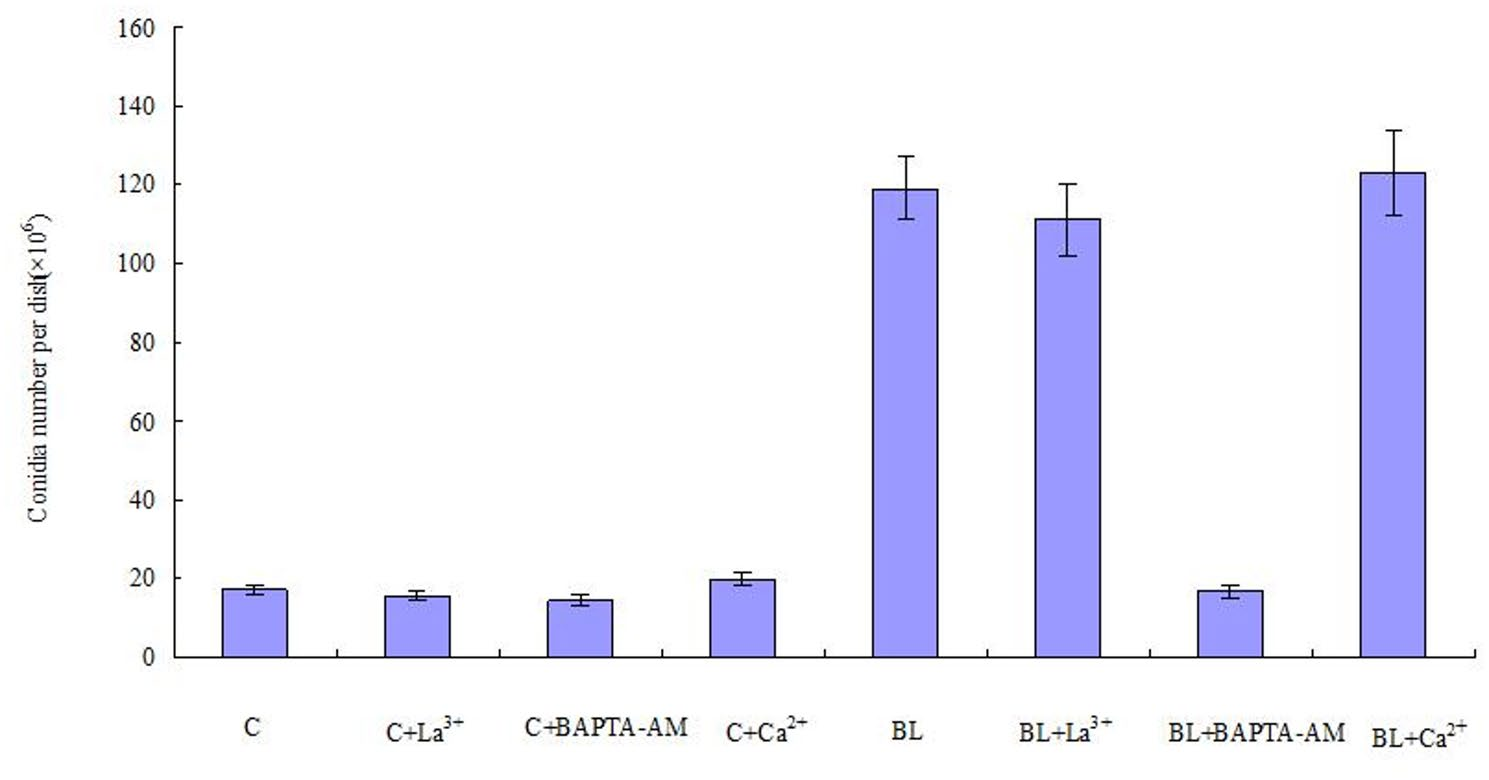
Effect of Ca^2+^ inhibitors on conidial production. *C* Control; *BL* Blue light. Values were means of five independent experiments. Bars represented standard errors

The DEPs showed that many proteins involved in Ca^2+^ signaling were differentially expressed. Blue light induced a significant increase of calcium ion binding (CIB) and calcium/calmodulin- dependent protein kinase (CaMK) in conidia. To investigate the signaling mechanism of conidial production induced by blue light, we investigated the effects of blue light on Ca^2+^ and conidial production. In Fig. 6, the effects of Ca^2+^ inhibitors LaCl_3_ and BAPTA-AM on blue light induced conidial production were studied. La^3+^ acts as an inhibitor of Ca^2+^ channels on the cell membrane and inhibits extracellular Ca^2+^ inflow (Gao et al. 2022). We chose to add La^3+^ to *S. bambusicola* grown on modified PDA medium for 4 days, and found that this did not affect the number of conidia (*P* > 0.05). However, treatment with BAPTA-AM not only blocked light-induced conidiation but also decreased the conidial production, and the yield was significantly reduced to 1.94 × 10⁵ conidia/cm² (*P* < 0.01). Intracellular calcium chelating agent BAPTA-AM inhibited conidial production, while plasma membrane calcium channel inhibitor LaCl_3_ did not affect conidial production. Therefore, it can be inferred that Ca^2+^ participate in regulating the production of conidia under the influence of blue light. In the process of producing conidia from mycelia of *S. bambusicola*, free Ca^2+^ do not enter cells through plasma membrane calcium channels, but from intracellular calcium pools. The addition of exogenous calcium resulted in a slight increase in conidial production, but there was no significant difference compared with that in the control group (*P* > 0.05). These results indicated that the input of exogenous calcium did not significantly change the conidial production when the mycelia produced conidia under blue light. Intracellular calcium pools are important signal sources of conidial production induced by blue light.

**Fig. 7:**
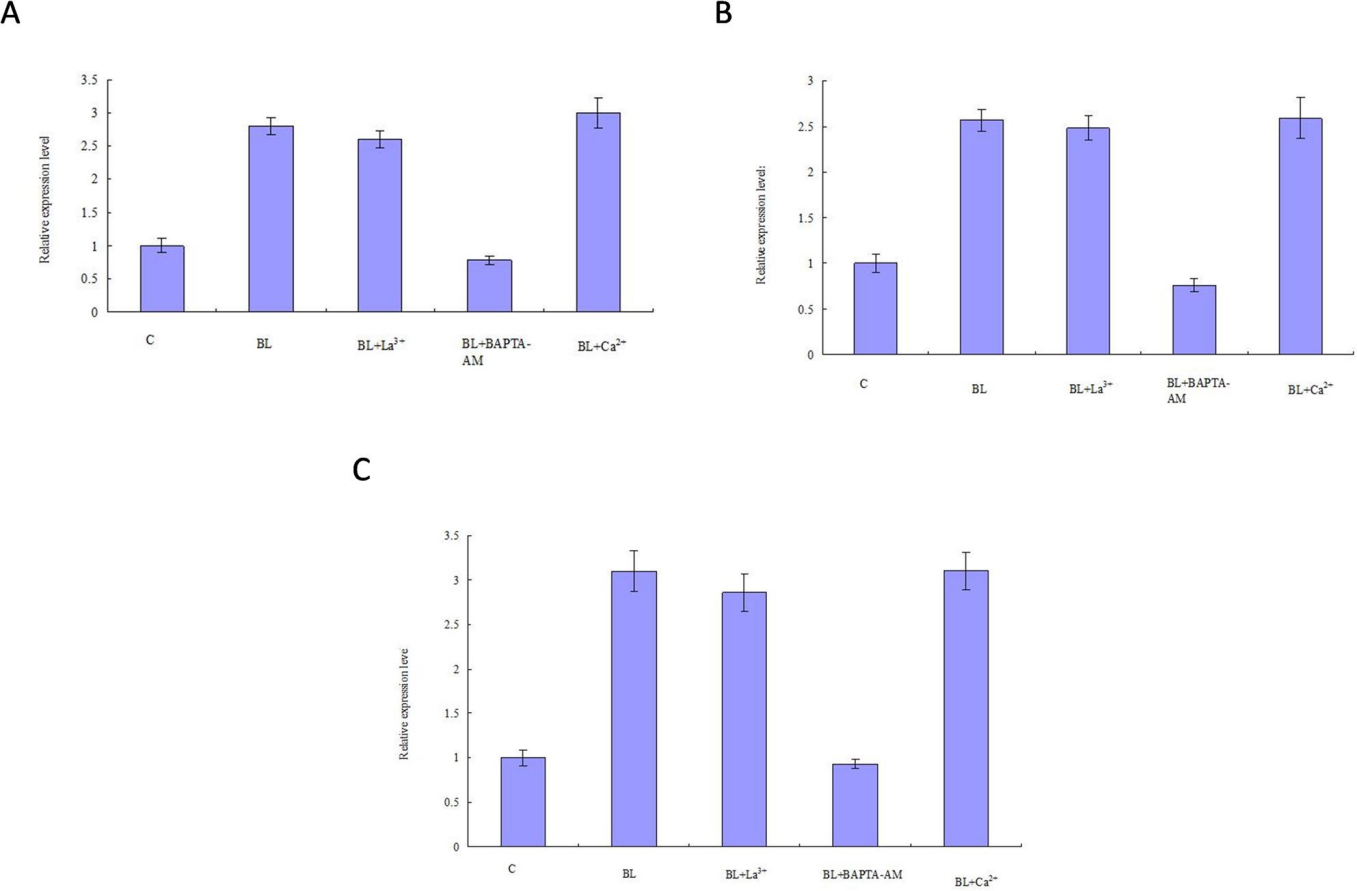
Quantitative PCR validations of genes. **A**: *CaMK*; **B**: *CANX*; **C**: *GH.* In the figure, C and BL represent Control and Blue Light, respectively. Values were means of five independent experiments. Bars represented standard errors

**Fig. 8:**
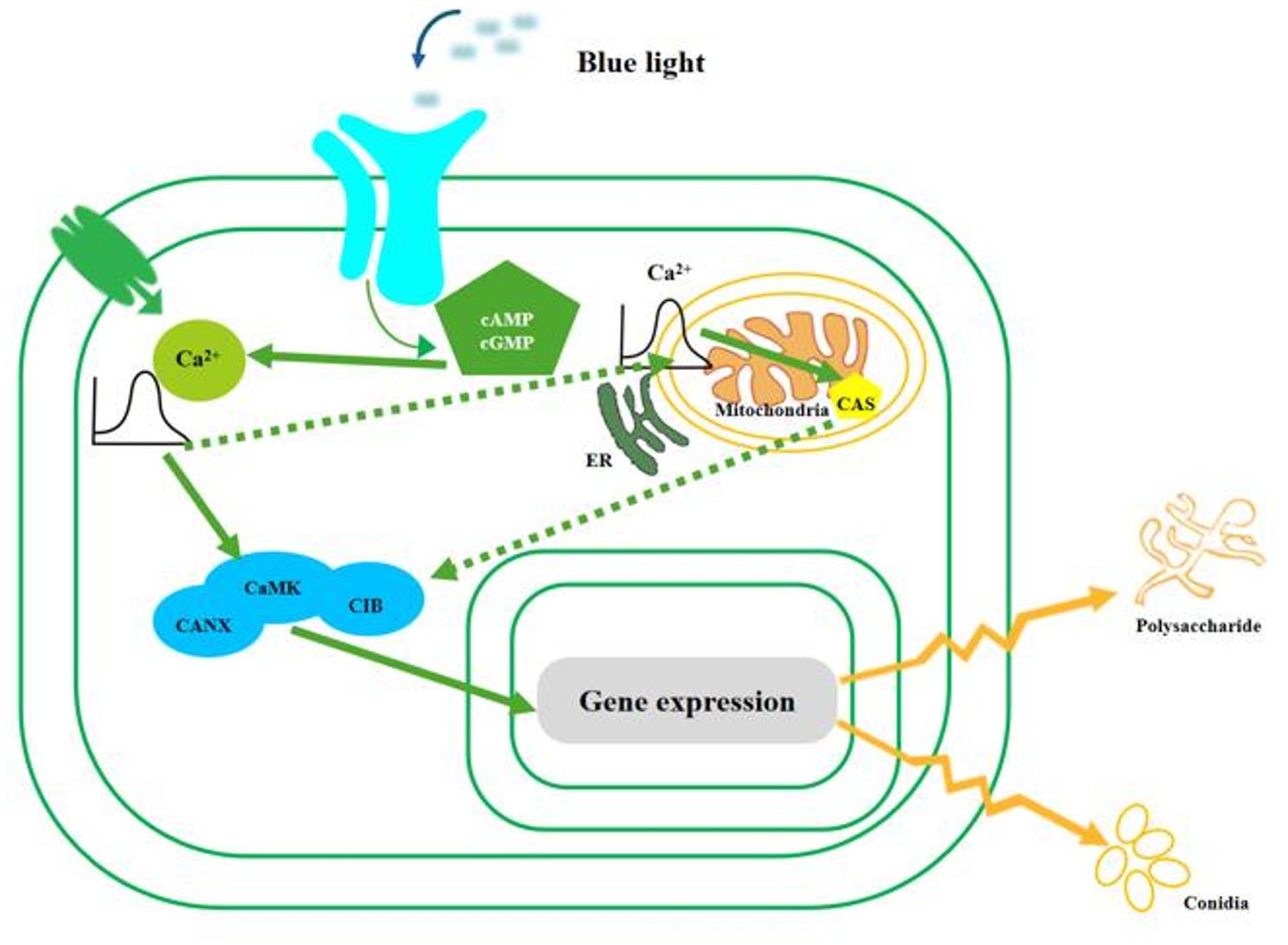
The signaling pathway on *S. bambusicola*-blue light interactions

The DEPs of conidial production in *S. bambusicola* showed that CANX, calreticulin (CRT), intracellular protein transport, serine/threonine-protein kinase (STK), CIB, CaMK were differentially expressed in the cellular regulation mediated by Ca^2+^ signal. According to the regulatory characteristics of Ca^2+^ signals and DEPs data, we selected the *CaMK* and *CANX* genes. *GH* gene was selected according to the DEPs during conidial formation. The effects of LaCl_3_, BAPTA-AM and Ca^2+^ on gene expression of key enzymes in conidial formation of *S. bambusicola* were studied. The results of qPCR showed that blue light could significantly up-regulate the expression of *CaMK*, *CANX* and *GH* in *S. bambusicola* cells. They exhibited significant increases of 2.81-, 2.57-, and 3.10-fold, respectively. These upregulations were statistically significant (*P* < 0.01) and were consistent with the proteomics data. The difference in specific results may be caused by different methods, detection indicators and translation efficiency. BAPTA-AM inhibited the promoting effect of blue light on *CaMK*, *CANX* and *GH* expression, significantly reducing their expression levels (*p* < 0.01), and affecting conidial production (Fig. 7). The effects of LaCl_3_ and Ca^2+^ on the expression levels of *CaMK*, *CANX* and *GH* were not significant (*p* > 0.05), which may be because of the presence of more Ca^2+^ in PDA medium, and extracellular Ca^2+^ was no longer required for the growth of conidia from mycelia of *S. bambusicola*. These results indicate that Ca^2+^ is a signaling molecule necessary for blue light to activate the conidial biosynthetic pathway, and further confirm that the intracellular calcium pools were the main source of Ca^2+^ signaling from the mycelia induced conidial production (Fig. 8). However, how cells mediate the Ca^2+^ signaling pathway to produce conidia remains to be studied.

## Discussion

Currently, our understanding of light-regulated growth and development in macrofungi is relatively systematic. Generally, the vegetative growth stage does not require light induction, whereas specific light qualities, especially blue light, have been proven to significantly promote asexual conidiation in various fungi. For example, blue light can inhibit mycelial growth in *Tuber borchii* and induce oidia formation in *Coprinopsis cinerea* (Sisti et al. 2022; Liu et al. 2022). This study validates the multiple effects of blue light on *S. bambusicola*, including enhanced membrane permeability, increased biomass, elevated protein content and glycosylation activity, and most effectively induced conidiation (Du et al. 2024). These findings support the prevailing view that light promotes conidia formation by triggering stress responses (Tisch and Schmoll 2010; Li et al. 2020). At the same time, significant differences exist in light requirements among different fungi. For instance, *Cordyceps militaris* prefers darkness for vegetative growth but depends on light for conidiation (Feng et al. 2021), further illustrating the complexity of light regulatory mechanisms. Based on the above patterns, this study adopted a two-stage strategy of “first promoting mycelial growth through dark culture, followed by blue light induction to promote conidiation.” This design follows fungal biological characteristics, ensuring sufficient mycelial expansion and material accumulation before introducing blue light as a developmental signal, thereby efficiently and specifically initiating the conidiation program. This approach achieves simultaneous optimization of conidia yield and quality. Compared to continuous illumination or chemical induction, this method optimizes resource allocation and the production workflow, significantly enhancing production efficiency and product consistency while effectively reducing unit costs. Furthermore, as a pollution-free and easily controllable physical induction method, blue light offers an environmentally friendly strategy with high application potential for industrial-scale conidial production. Future research should focus on the precise optimization of process parameters to facilitate its large-scale application.

How does blue light stimulate a significant increase in conidial formation and metabolic activity in *S. bambusicola*? In this study, the proteome of *S. bambusicola* in the dark and the conidia formed under blue light was determined. On this basis, the GO annotation, KEGG pathway analysis, COG annotation and enrichment analysis of the DEPs were performed, which revealed that the reprogramming of carbohydrate metabolism, dynamic remodeling of cellular architecture, and concomitant stress responses collectively drive the sporulation process. Notably, the activation of the Ca²⁺ signaling pathway plays a central and integrative regulatory role in this process.

GO analysis revealed that during the blue light-induced sporulation process, the number of DEPs in intracellular non-membrane-bounded organelle and intracellular organelle lumen of CC was significant. It is involved in many metabolic processes. The DEPs are represented by chitin synthase (CHS1) and GH, which are involved in cell periphery, organic substance metabolic process. Beta-tubulin in cytoskeleton, tubulin alpha chain, endoplasmic reticulum chaperone BiP, vesicle-mediated transport, these proteins are significantly reduced. It may represent a reduction in the cellular components required for conidiation of *S. bambusicola* under blue light, suggesting that the mycelial morphology of conidia produced in blue light may be simpler than that in dark. It has also been shown that beta-tubulin contributes to cellular transport of organelles and cell wall materials, impacting growth, appressorial differentiation in *Metarhizium acridum* (Zhang et al. 2017a, b). In GO annotated BP analysis, carbohydrate metabolic process was the most significantly enriched term (*P* < 0.001), consistent with the COG annotation and enrichment results. The process of blue light promoting the conidiospore formation of *S. bambusicola* is a complex biological process, which may involve many biological pathways such as macromolecular assembly, small molecule metabolism, substance transport, and stimulus response. Among them, response to stimulus also has a high significance, and many significantly increased DEPs help maintain subcellular structures and protect biological macromolecules, remove ROS, and reduce damage (Tan et al. 2023). Compared with mycelia, these proteins were significantly up-regulated in the conidia-bearing mycelia of *S. bambusicola* BZ-16 under blue light, indicating that the cell is in a state of stress when mycelia differentiate to form conidia, producing many heat shock proteins and antioxidant enzymes, similar findings have been found in many macrofungi (Fu et al. 2022). MF analysis indicated that oxidative stress may play a role in blue light. There was significant difference in katG. This enzyme exhibits a greater affinity for H_2_O_2_ than the peroxidases found in animal cells, and it is characterized by exceptional enzymatic activity. It is involved in respiration, auxin oxidation, and other processes (Li et al. 2023). Compared with the control group, one katG was up-regulated and three katGs was down-regulated. It shows that they may belong to two distinct types and play the biological function of oxidation. The enzyme activity changes throughout the fungal growth and development stages, generally being greater in aging tissues and lower in younger tissues (Li et al. 2023; Derrien et al. 2023). Studies have demonstrated that an increase in peroxidase activity in the roots of prematurely aged rice reflects the potential role of peroxidase as a physiological indicator of tissue aging (Derrien et al. 2023).

In KEGG pathway, GH and trehalase in conidia under blue light increased in the starch and sucrose metabolism pathways, suggesting that the whole process may lead to a decrease in glycogen content. However, the amount of D-glucose increased and the degree of branching of glycogen decreased. Their alterations lead to changes in the structure and content of the polysaccharide products. This class of differential proteins suggests that a certain glycoside is required for the tissue components of the conidia to promote hydrolytic activity (Saito et al. 2023). The phagosome pathway showed high significance in the KEGG.Org analysis. Phagocytosis is essential for maintaining cellular homeostasis, regulating signal transduction and nutrient uptake, and is necessary for eukaryotic life activities (Liu et al. 2023). Clathrin-mediated phagocytosis is a highly conserved cellular process. Studies have shown that protein transport protein Sect. 61 subunit alpha, calnexin-like protein, CRT, vacuolar proton pump subunit B. were significantly increased. CANX is a calcium-binding protein in the endoplasmic reticulum or sarcoplasmic membrane that is involved in regulating the proper folding of proteins. It plays an important role in regulating the cytosolic free calcium concentration in *Aspergillus nidulans*, which is closely related to the formation of conidia and the growth of hyphae (Zhang et al. 2017a, b). The Sect61 complex is a key element in the protein transport mechanism and, MoSec61β plays an important role in the hypha morphology, sporulation and pathogenicity of *Magnaporthe oryzae* (Wei et al. 2020). In the species-specific KEGG analysis, the phagosome pathway in *A. alternata* exhibited significant differential enrichment (*P* = 0.0378). Significantly altered genes in this pathway included vacuolar proton pump subunit B, calreticulin, beta-tubulin (fragment), protein transport protein Sect. 61 subunit alpha and other related proteins. In the COG database, carbohydrate transport and metabolism proteins showed the most significant differences. There was significant expression of MAN1C1, beta-hexosaminidase and other proteins involved in glycan biosynthesis. MAN1C1 belongs to the glycosyl hydrolase family 47. In lower eukaryotes such as *Saccharomyces cerevisiae* only contain MAN1C1 in the endoplasmic reticulum and synthesize high-mannose N-glycans, it is associated with the dimorphism of *Sporothrix schenckii* (Kumakura et al. 2019).

Ca^2+^ is an important intracellular messenger widely existing in cells, and almost all cell physiological activities are regulated by Ca^2+^. During fungal growth, Ca^2+^ not only affects cell permeability, but also affects cell absorption of phosphate in the medium and the polarization growth of mycelia. Ca^2+^ concentration in the medium has an effect on the branching probability of many fungal mycelia (Roy et al. 2020). It is particularly noteworthy that in *Trichoderma*, a rapid increase in cytosolic Ca²⁺ concentration is crucial for translating the physical damage stimulus into a coordinated conidiation response (Macías-Rodríguez et al. 2020; Wang et al. 2024). The dependence of conidiation on Ca²⁺ signaling is observed across various induction mechanisms—including physical damage in *Trichoderma* and the blue light induction examined in this study. This suggests that the Ca²⁺ signaling module is a common central pathway through which fungi perceive environmental stimuli to trigger asexual development. It was found that during the process of conidia formation in mycelia of *S. bambusicola* under blue light illumination, the related proteins of Ca^2+^ signal changed significantly. Previous studies have identified several calcium-signaling related proteins, such as CIB, CaMK, Calponin homology domain-containing protein, Calreticulin (CRT), CANX, Tricalbin, and Calmodulin. The qPCR study found that BAPTA-AM inhibited the promoting effect of blue light on the expression of *CaMK*, *CANX*, *GH*, and significantly reduced their expression levels. The effects of LaCl_3_ and Ca^2+^ on the expression levels of *CaMK*, *CANX* and *GH* were not significant. The results indicated that the conidial production was induced by the intracellular calcium pools (Fig. 8). The regulation of calcium signaling in the solute is mainly controlled by Ca^2+^ uptake or release from the cellular calcium pool. The endoplasmic reticulum and mitochondria are the most important calcium pools in the cell and play an extremely important role in calcium signaling. Current studies believe that intracellular calcium homeostasis is mainly maintained through the endoplasmic reticulum, which can store most Ca^2+^ (Li et al. 2021). In eukaryotic microorganisms, Ca^2+^ signaling pathway has an important impact on the growth and development of pathogenic fungi, especially their virulence and infectivity (Roy et al. 2020). For example, the absence of *CaMK* gene in *Arthrobotrys oligospora* can reduce the ability of cells to produce conidia and traps, causing a deficiency in nematicidal ability as well (Roy et al. 2020; Li et al. 2021, 2022). *PiCaMK1* plays a key role in the regulation of multiple physical and cellular processes including growth, conidiation, virulence and environmental stress tolerance of the pathogen *Penicillium italicum* (Li et al. 2022). Blue light can regulate the growth and development of macrofungi and the production of metabolites. It has been proved that light can affect the pathogenicity of a variety of pathogenic fungi (Shad and Shad 2021), so whether this function has a similar function in large fungi such as *S. bambusicola* deserves further study.

## Conclusions

This study employed a two-stage cultivation strategy of “dark incubation followed by blue light induction,” which confirmed the significant enhancement of conidial production in *S. bambusicola* by blue light. This process was accompanied by profound proteomic responses, primarily manifested as structural remodeling of intracellular organelle lumens and non-membrane-bounded organelles. DEPs were significantly enriched in pathways related to carbohydrate transport and metabolism, as well as the phagosome, indicating that conidiation relies on active carbon source utilization, material transport, and cellular autophagy to achieve energy supply and structural reconstruction. The study further confirmed that the Ca²⁺ signaling pathway plays a central regulatory role in this process, and its activation is a critical step in blue light-induced conidiation. In summary, blue light efficiently initiates the conidiation program in *S. bambusicola* by coordinately driving carbohydrate metabolic reprogramming, dynamic remodeling of cellular structures, and Ca²⁺ signal transduction. This work systematically elucidates the multidimensional molecular mechanisms by which blue light regulates conidial formation and provides an important theoretical basis and practical strategy for the environmentally friendly and large-scale production of fungal conidia through physical induction.

## Supplementary Information

Below is the link to the electronic supplementary material.

Supplementary Material 1.

## Data Availability

No datasets were generated or analysed during the current study.
