# Supplementary material for: Blue light regulates conidia production in Shiraia bambusicola via calcium/calmodulin signaling
Source: AMB Express. 2026 Mar 7;16:43. doi: 10.1186/s13568-026-02036-2 (PMC13079248; doi:10.1186/s13568-026-02036-2)

Table S1 Primers for the gene transcription level analysis. F: forward primer, R: reverse primer.

Genes	Gene description	Name of primer	Sequence
18S	Reference gene	18S-F	5'-ACGCAGCGAAATGCGATAAG-3'
		18S-R	5'-CAAATTGTGCTGCGCTCCAA-3'
GH	Glycosyl hydrolase	GH-F	5'-CGATAACTCTGGCAAGTACGG-3'
		GH-R	5'-AAGGACTTTGATGGTGTAGGC-3'
CANX	Calnexin	ClxA-F	5'-GTAGATGGGAAGCCTCGATTG-3'
		ClxA-R	5'-TGACTACCTGAAATCCAAACCG-3'
CaMK	Calcium/calmodulin-dependent protein kinase	CaMK-F	5'-CTATCCCACACCATTCATCCC-3'
		CaMK-C	5'-TCGTCAGCCTTGTCTTCATC-3'

Fig. S1 Distribution of peptide mass deviation

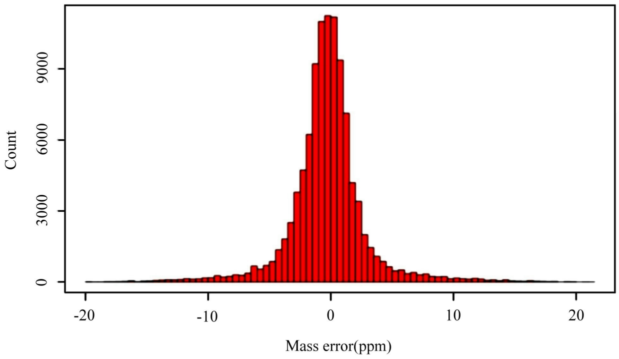

Fig. S2 The number of missed cleavages

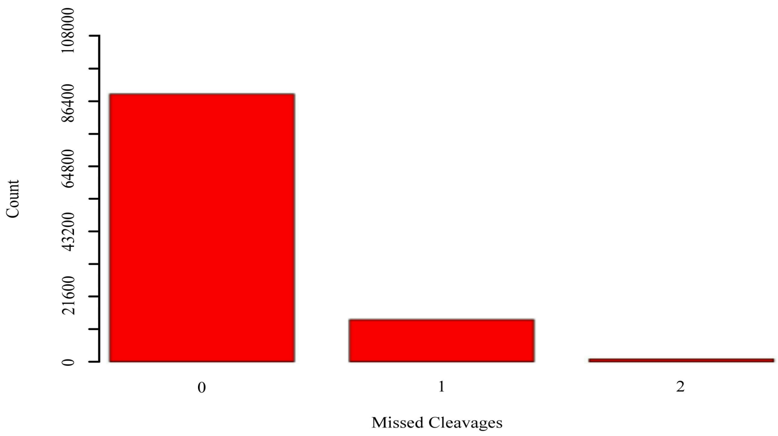

Fig. S3 Conidia of *S. bambusicola* under optimal yield

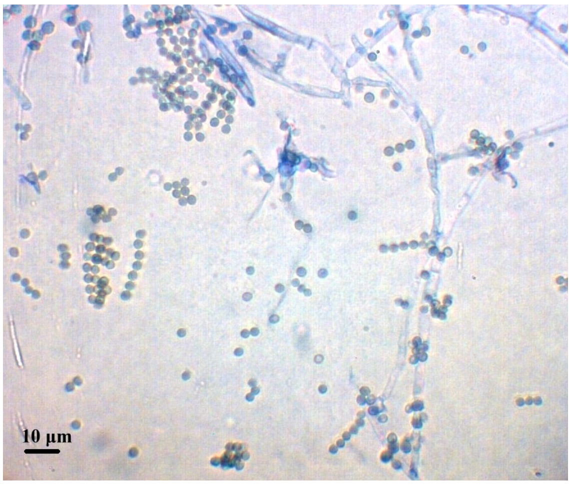

Supplement: Supplementary file 1 — Supplementary Material 1. [file 13568_2026_2036_MOESM1_ESM.pdf]
